# Survival Benefits for Pulmonary Adenocarcinoma With Malignant Pleural Effusion After Thoracoscopic Surgical Treatment: A Real-World Study

**DOI:** 10.3389/fonc.2022.843220

**Published:** 2022-05-05

**Authors:** Xin Li, Mingbiao Li, Jinshuang Lv, Jinghao Liu, Ming Dong, Chunqiu Xia, Honglin Zhao, Song Xu, Sen Wei, Zuoqing Song, Gang Chen, Hongyu Liu, Jun Chen

**Affiliations:** ^1^Department of Lung Cancer Surgery, Tianjin Medical University General Hospital, Tianjin, China; ^2^Tianjin Key Laboratory of Lung Cancer Metastasis and Tumor Microenvironment, Tianjin Lung Cancer Institute, Tianjin Medical University General Hospital, Tianjin, China; ^3^Department of Thoracic Surgery, First Affiliated Hospital, School of Medicine, Shihezi University, Shihezi, China

**Keywords:** malignant pleural effusion, pulmonary adenocarcinoma, video-assisted thoracoscopic surgery, pleurodesis, chemotherapy

## Abstract

**Objectives:**

Malignant cells in the pleural fluid or pleural metastasis are classified as stage IV non-small cell lung cancer. Radical surgery is generally considered not suitable for such patients. The aim of our study was to discuss the effectiveness of video-assisted thoracoscopic surgery (VATS) in such patients.

**Methods:**

A retrospective analysis of the clinical records of 195 patients was performed. These patients were all diagnosed with locally advanced pulmonary adenocarcinomas with malignant pleural effusion (MPE, M1a) but no distant organ metastasis. The 195 patients included 96 patients who underwent VATS plus chemotherapy and 99 patients who received thoracic drainage plus chemotherapy. The baseline characteristics of the patients included age, gender, smoking history, Eastern Cooperative Oncology Group (ECOG) score, and number of chemotherapy cycles (2–4 cycles or >4 cycles); we also analyzed clinical characteristics including the specific surgical options of the VATS group.

**Results:**

In multivariate analysis, when compared to the thoracic drainage group, the VATS group remained significantly associated with the overall survival [HR=0.480 (95%CI 0.301-0.765)]; when compared to the lobectomy, the sub-lobectomy and the palliative surgery, remained significantly associated with the overall survival [HR=0.637 (95%CI 0.409-0.993) and HR=0.548 (95%CI 0.435-0.832), respectively]. The median survival time (MST) of patients who underwent VATS (n = 96, 49.2%) was 25 months (95% CI 22.373–27.627) whereas the patients who received thoracic drainage (n = 99, 50.8%) was 11 months (95% CI 9.978–12.022). For patients who underwent VATS, the MST of patients who received a lobectomy (n = 50, 52.1%) was 27 months (95% CI 22.432–31.568), the MST of patients who received a sub-lobectomy plus pleurodesis (n = 26, 27.1%) was 27 months (95% CI 19.157–34.843), and the MST of patients who received only pleurodesis (n = 20, 20.8%) was 12 months (95% CI 7.617–16.383).

**Conclusion:**

For pulmonary adenocarcinomas with MPE, receiving a lobectomy or sub-lobectomy plus pleurodesis with VATS was associated with improved survival compared with patients who only received thoracic drainage and chemotherapy. Our results and previously published data may justify the use of VATS for treating pulmonary adenocarcinomas with MPE.

## Introduction

Recently, lung cancer has been shown to have the highest death rate of all types of malignant tumors worldwide (1, 2). Non-small cell lung cancer (NSCLC) accounts for 75%–80% of all lung cancers, of which 50% are adenocarcinomas. Parietal pleural metastasis often occurs in advanced-stage adenocarcinoma and often develops into malignant plural effusion (MPE). Although many malignant tumors can cause MPE, adenocarcinoma is the most common pathological type, accounting for 45%–65% of all pathological types (3). Although treatment for NSCLC is constantly improving, the sensitivity of MPE to the current existing treatment methods is poor, which leads to a higher mortality rate. Palliative treatment is often used for treating such patients; however, even with systemic chemotherapy, these patients only have an overall median survival time (MST) of 3–12 months (4, 5). Existing treatment methods for such patients include thoracentesis, pleurodesis, thoracic drainage, chemotherapy, radiotherapy, anti-angiogenesis therapy, targeted therapy, etc. MAY most people consider chemotherapy or targeted therapy after thoracic drainage as appropriate for NSCLC tumors with MPE, but patients with good performance and only local metastasis may have a better quality of life and life expectancy after undergoing more aggressive therapies such as thoracoscopic surgery (6). Several studies have indicated that surgical therapy may provide a survival benefit to specific subsets of NSCLC patients with MPE (7–9). Therefore, in this study, we attempted to explore whether chemotherapy or targeted therapy after video-assisted thoracoscopic surgery (VATS) can improve the prognosis of NSCLC patients with MPE compared with those receiving a traditional treatment method.

## Methods

### Patients and Groups

After screening, 195 primary lung adenocarcinoma patients with MPE but no distant organ metastasis were admitted to the Tianjin Medical University General Hospital from January 2009 to March 2015. Of these patients, 96 underwent chemotherapy or targeted therapy after VATS (VATS group), and 99 underwent traditional treatment (chemotherapy after thoracic catheterization; thoracic drainage group). Patient demographic information and clinical pathology data were collected. This clinical study was approved by the Ethics Committee of our institute. Preoperatively, the patients received a thorough physical examination and blood examination, respiratory function test, electrocardiogram, bone emission computed tomography (ECT), bronchoscopy, brain magnetic resonance imaging (MRI), and computed tomography (CT) of the chest and abdomen. Preoperative biopsy and intraoperatively biopsy confirmed that all patients had locally advanced stage IV disease.

The OS of the VATS group was defined as the time from the beginning of surgery to death from any cause. The OS of the thoracic drainage group was defined as the time from when the thoracic drainage tube was placed in the patients to death from any cause. For patients who were still alive at the end of data entry, the time of the last follow-up or medical record of the patient was taken as the cut-off time.

The baseline characteristics of the patients included age, gender, smoking history, ECOG score, and number of chemotherapy cycles (2–4 or >4 cycles). The other clinical characteristics we analyzed included the specific surgical options of the VATS group. The different surgical methods of patients in the VATS group were distributed as follows: 50 patients (52.1%) received lobectomy plus pleurodesis and were classified as the “lobectomy subgroup,” 26 patients (27.1%) received segment or wedge resection plus pleurodesis and were classified into the “sub-lobectomy subgroup,” and 20 patients (20.8%) received only pleurodesis under thoracoscope and were classified into the “palliative surgery subgroup.”

All patients (100%) were followed-up in our study. Information was obtained from all patients through outpatient visits or telephone calls. All patients were evaluated every three months by chest and abdominal CT scans and brain MRI, and ECT was performed every six months for the first two years after surgery and annually thereafter. Overall survival (OS) was estimated from the date of lung surgery or thoracic drainage or until the last follow-up.

### Therapy Procedure

All patients were diagnosed with primary lung adenocarcinoma with MPE from tumor cells that were found in the pleural effusion and were then diagnosed with adenocarcinoma by bronchoscopy or percutaneous lung puncture.

In the VATS group, the primary tumors were considered resectable when the patients had a good Eastern Cooperative Oncology Group (ECOG) score and no severe comorbidities. Complete resection and limited resection were defined as lobectomy and sub-lobectomy (wedge or segment resection), respectively. All pleural metastatic lesions were removed or cauterized with a high frequency electric knife as much as possible. After that, pleurodesis was performed with 1% iodine tincture under thoracoscopy. Those tumors that could not be resected were given only pleurodesis with 1% iodine tincture under thoracoscopy after all pleural metastatic lesions were cauterized with a high frequency electric knife. All the 2mm sizes visible under the microscope are treated. One to two weeks after surgery, the patients were given two cycles of chemotherapy (pemetrexed or paclitaxel plus platinum).

**Figure 1:**
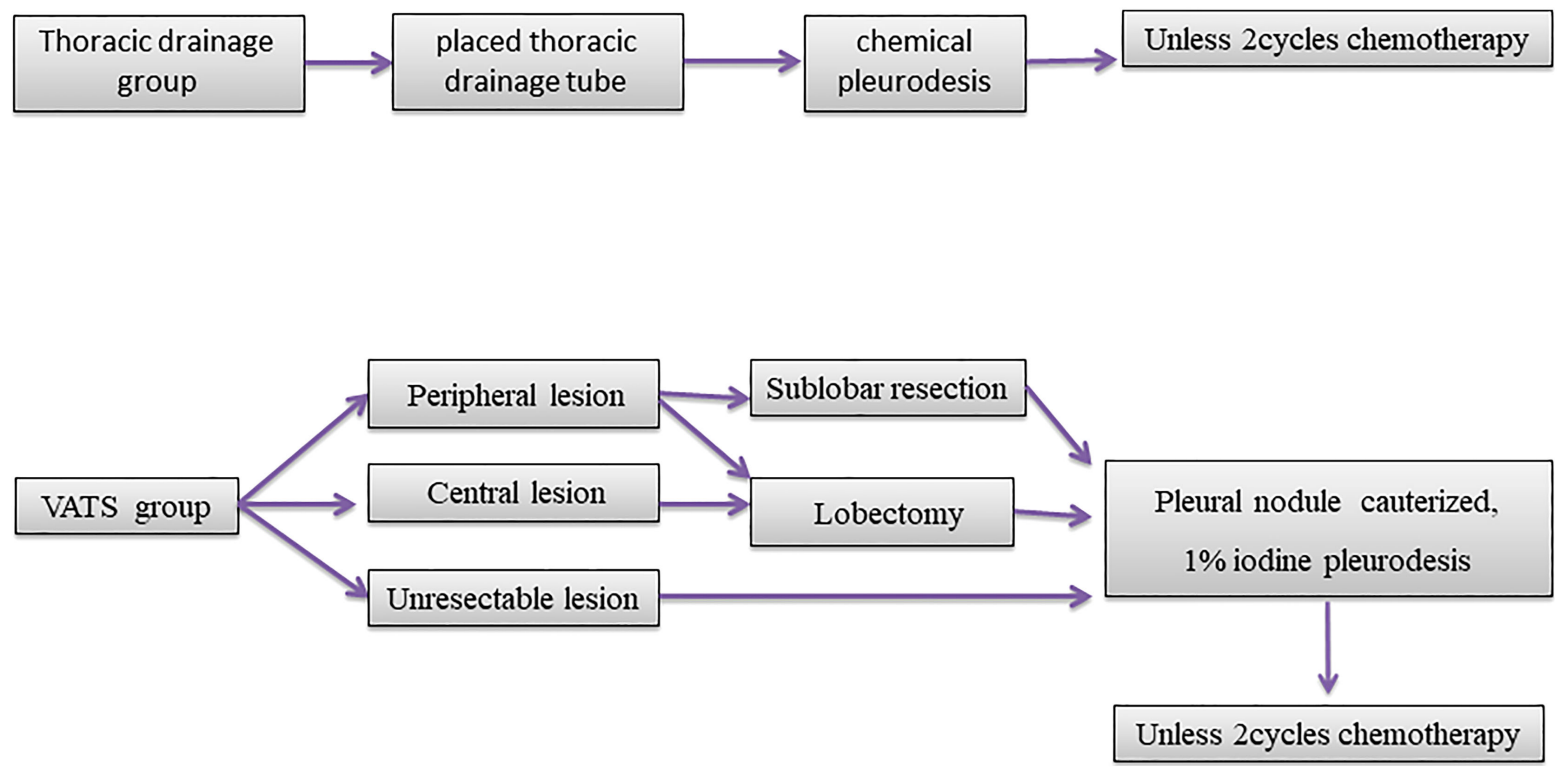
Outline of two groups of patients with different treatment process.

In the thoracic drainage group, a thoracic drainage tube was placed in the patients under the guidance of ultrasonograhy. After the effusion was drained, adhesive, sclerosing agent, or cisplatin were injected into the chest cavity for chemical pleurodesis. The patients were given two cycles of chemotherapy (pemetrexed or paclitaxel plus platinum). The dosage and administration of chemotherapy drugs that all patients received were recommended by the National Comprehensive Cancer Network (10). None of the patients had received neoadjuvant chemotherapy, radiotherapy, or targeted therapy before surgery or thoracic drainage (**Figure 1**).

### Efficacy Evaluation Criteria

The World Health Organization’s unified criteria of MPE efficacy defines the responses to MPE treatment as follows: complete response (CR), in which the MPE completely disappeared and symptoms were completely relieved, which was maintained for more than four weeks; partial response (PR), in which the MPE volume was significantly reduced by >50% and the symptoms were obviously relieved for more than four weeks; and no change (NC), in which the above criteria were not met or the MPE was increased a short time after the reduction. The response rate (RR) is defined as CR + PR.

### Statistical Analysis

All data were processed by SPSS 17.0 statistical software. Multivariate Cox regression models were used to assess the association between treatment and OS. The Cochran-Mantel-Haenszel (CMH) X^2^ test was used to evaluate the treatment effect on MPE between the VATS group and the thoracic drainage group, and the Kaplan–Meier method was used to analyze the OS of the two groups and subgroups. The difference was statistically significant when p < 0.05.

## Results

The median age of all patients was 61.6 years old. There were 113 male patients (57.9%) and 82 female patients (42.1%); 104 patients (53.3%) had a smoking history and 91 patients (46.7%) had no smoking history; 79 patients (40.5%) had an ECOG score of 0–1, and 116 patients (59.5%) had an ECOG score of 2 or higher. The baseline characteristics of the two groups are shown in **Table 1**. For the baseline characteristics of age (p = 0.361), gender (p = 0.328) and smoking history (p = 0.954), there were no significant differences between the two groups; however, there was a significant difference in ECOG score (p < 0.05). Specifically, 53.1% of patients in the VATS group had an ECOG score of 0–1, which was significantly higher than the 28.3% in the thoracic drainage group. In multivariate analysis, when compared to the thoracic drainage group, the VATS group remained significantly associated with the overall survival [HR=0.480 (95%CI 0.301-0.765)]; when compared to the lobectomy, the sub- lobectomy and the palliative surgery, remained significantly associated with the overall survival [HR=0.637 (95%CI 0.409-0.993) and HR=0.548 (95%CI 0.435-0.832), respectively] (**Tables 2**, **3**).

**Table 1:** Baseline characteristics of all patients.

		VATS group(n = 96)	Thoracic drainagegroup (n = 99)	p value
	Median age	61	62.3	
Age (years)	≤60	46 (47.9%)	41 (41.4%)	0.361
	>60	50 (52.1%)	58 (58.6%)	
Gender	Male	59 (61.5%)	54 (54.5%)	0.328
	Female	37 (38.5%)	45 (45.5%)	
Smoking status	Non smoker	45 (46.9%)	46 (46.5%)	0.954
	smoker	51 (53.1%)	53 (53.5%)	
ECOG	0-1	51 (53.1%)	28 (28.3%)	<0.01
	≥2	45 (46.9%)	71 (71.7%)	
Number of chemotherapy cycles	2-4	38 (39.6%)	38 (38.4%)	0.864
	>4	58 (60.4%)	61 (61.6%)	
Surgical options	Lobectomy	50 (52.1%)	/	/
	Sub-lobectomy	26 (27.1%)	/	/
	palliative surgery	20 (20.8%)	/	/

**Table 2:** Multivariate Cox model for overall survival in all patients.

		Case (%)	HR (95%CI)	p value
Case		199 (100%)		
Treatment mode	VATS group	96 (49.2%)	0.480 (0.301-0.765)	0.002
	Thoracic drainage group	99 (50.8%)	–	–

**Table 3:** Multivariate Cox model for overall survival in the VATS group.

		Case (%)	HR (95%CI)	p value
Case		96 (100%)		
Treatment mode	Lobectomy	50 (52.1%)	0.637 (0.409-0.993)	<0.01
	Sub- lobectomy	26 (27.1%)	0.548 (0.435-0.832)	0.001
	Palliative surgery	20 (20.8%)	–	–

### Analysis of MPE Therapeutic Effect in the Two Groups

**Figure 2:**
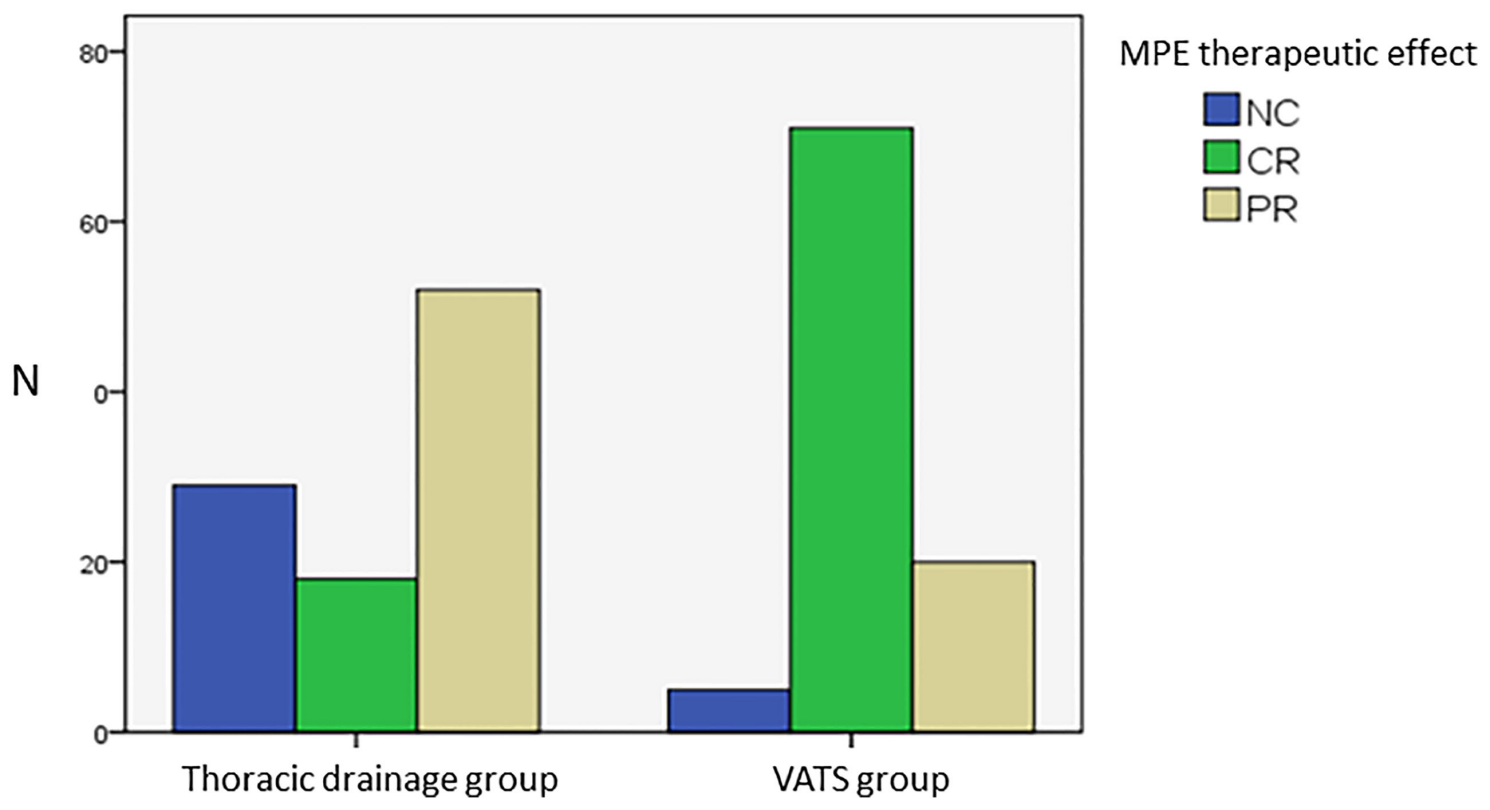
Analysis of MPE therapeutic effect of two groups.

The therapeutic effects of the two groups for MPE were distributed as follows: VATS group: NC (5 cases, 5.2%), CR (71 cases, 74%), and PR (20 cases, 20.8%), with an RR of 94.8%; thoracic drainage group: NC (29 cases, 29.3%), CR (18 cases, 18.2%), and PR (52 cases, 52.5%), with an RR of 70.7%. The CMH X^2^ test was used to assess whether there was any difference in MPE efficacy between the two groups. The location test results had a p of 0.0037, rejecting the hypothesis of H_0_; thus, the effect differed between the two groups, and the difference was statistically significant. In conclusion, according to the row average score, the efficacy for treating MPE in the VATS group was better than that in thoracic drainage group (**Figure 2**, **Table 4**).

**Table 4:** Comparison of the MPE therapeutic effect between the two groups.

MPE therapeutic effect	VATS group	Thoracic drainage group	p value
CR	71 (74%)	18 (18.2%)	0.0037
PR	20 (20.8%)	52 (52.5%)	
NC	5 (5.2%)	29 (29.3%)	

### Survival Analysis

**Figure 3:**
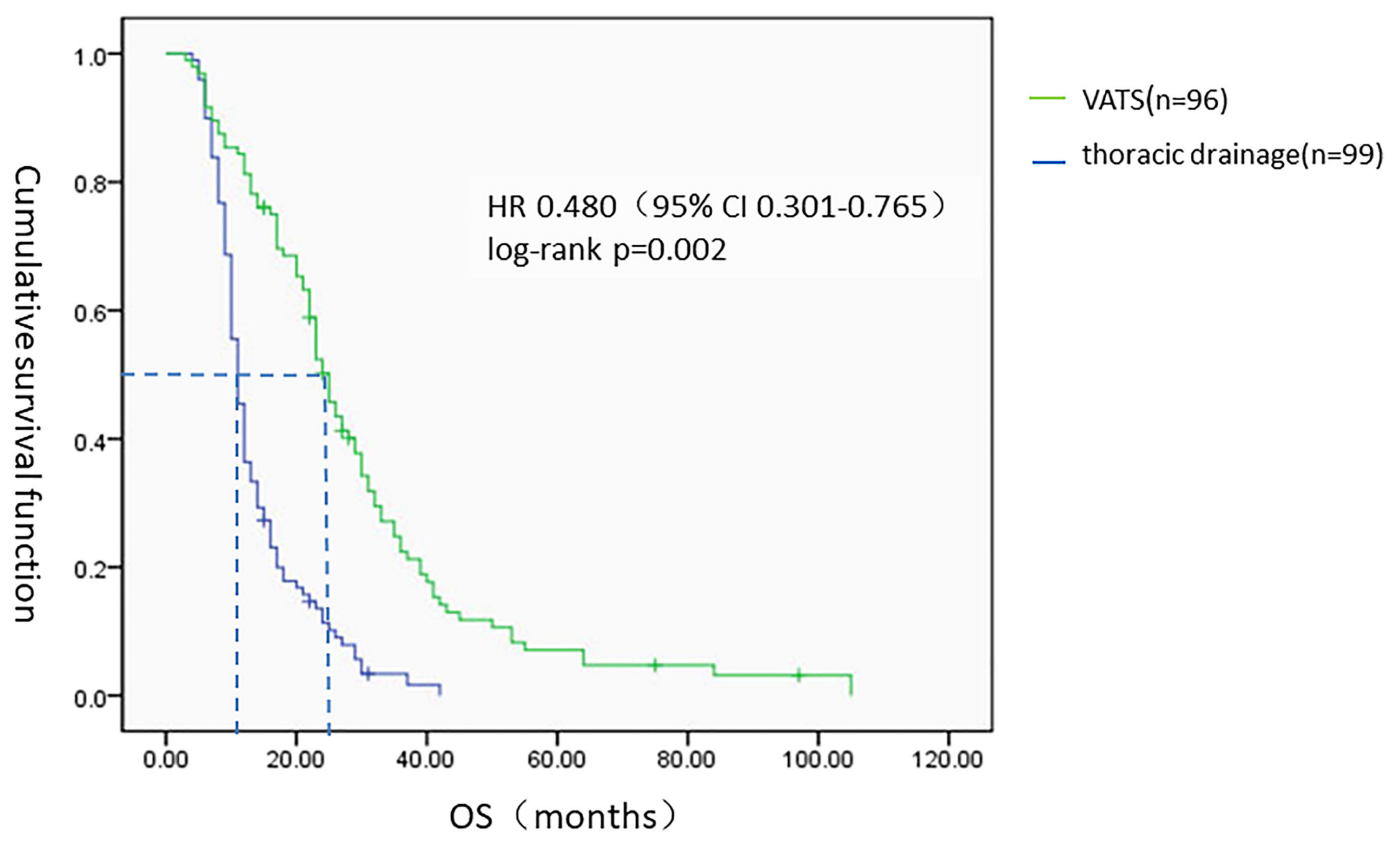
The Kaplan–Meier survival analysis of two groups. HR, hazard ratio.

All 195 patients were followed-up to the last follow-up time (March 2018). The MST from the onset of primary lung adenocarcinoma with MPE to death from any cause was 16 months [95% confidence interval (95% CI) 13.439–18.561]. For the 96 patients in the VATS group (49.2%), the MST was 25 months (95% CI 22.373–27.627), and the one-year and three-year survival rates were 88.6% and 21.6%, respectively. For the 99 patients (50.8%) in the thoracic drainage group, the MST was 11 months (95% CI 9.978–12.022), and the one-year and three-year survival rates were 36.4% and 1%, respectively. The hazard ratio (HR) was 0.480 (95% CI 0.301–0.765, log-rank p = 0.002). The MST in the VATS group was much longer than that in the thoracic drainage group (**Figure 3**). Generally, the Kaplan–Meier method was used to analyze the survival of all subgroups. In all subgroups, the survival benefit of the VATS group was significantly better than that of the thoracic drainage group, as indicated by the statistically significant log-rank test results (log-rank p < 0.01) (**Tables 5**, **6**).

**Table 5:** Survival analysis of all subgroups.

		VATSMST (months) (95%Cl)	Thoracic drainageMST (months) (95%Cl)	p
Age	≤60	23 (20.610-25.390)	11 (9.435-12.565)	<0.01
	>60	25 (20.541-29.459)	11 (9.763-12.237)	<0.01
Gender	Male	23 (19.872-26.128)	10 (9.042-10.958)	<0.01
	Female	31 (20.410-41.590)	12 (9.809-14.191)	<0.01
Smoking history	No	29 (22.205-35.795)	15 (10.373-19.627)	<0.01
	Yes	23 (21.060-24.940)	10 (9.112-10.888)	<0.01
ECOG	0-1	30 (24.533-35.467)	20 (14.979-25.021)	<0.01
	≥2	17 (11.920-22.080)	10 (9.323-10.677)	<0.01
Number of chemotherapy cycles	2-4	23 (13.364-32.636)	9 (7.792-10.208)	<0.01
	>4	26 (21.027-30.973)	13 (10.915-15.085)	<0.01

**Table 6:** Survival analysis of VATS group.

		N (%)	MST (months) 95%Cl	p value
VATS group		96 (100%)	25 (22.373-27.627)	
Age(years)	≤60	46 (47.9%)	23 (20.610-25.390)	0.292
	>60	50 (52.1%)	25 (20.541-29.459)	
Gender	male	59 (61.5%)	23 (19.872-26.128)	0.172
	female	37 (38.5%)	31 (20.410-41.590)	
Smoking history	No	45 (46.9%)	29 (22.205-35.795)	0.001
	Yes	51 (53.1%)	23 (21.060-24.940)	
ECOG	0-1	51 (53.1%)	30 (24.533-35.467)	<0.01
	≥2	45 (46.9%)	17 (11.920-22.080)	
Number of chemotherapy cycles	2-4	38 (39.6%)	23 (17.449-28.551)	0.311
	>4	58 (60.4%)	26 (21.027-30.973)	
Surgical options	1.lobectomy	50 (52.1%)	27 (22.432-31.568)	1 vs 2 0.915
	2.Sub- lobectomy	26 (27.1%)	27 (19.157-34.843)	2 vs 3 0.001
	3.palliative surgery	20 (20.8%)	12 (7.617-16.383)	1 vs 3 <0.01

**Figure 4:**
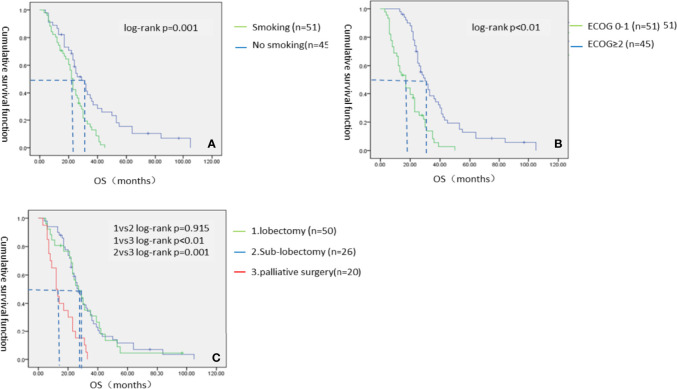
The Kaplan–Meier survival analysis of VATS groups. **(A)** Smoking status; **(B)** ECOG score; **(C)** Specific surgical options of VATS group.

These results indicate that patients in the VATS group have better survival benefits than those in the thoracic drainage group. The question then arises as to which factors can lead to better survival benefits in the VATS group. The Kaplan–Meier method was used to analyze the survival of all subgroups in the VATS group. We found that no smoking history, an ECOG score of 0–1, and undergoing a lobectomy or sub-lobectomy significantly improved OS in the VATS group. When stratified by patients who underwent different surgical options, we found that patients in the lobectomy subgroup had an MST of 27 months (95% CI 22.432–31.568), patients in the sub-lobectomy subgroup had an MST of 27 months (95% CI 19.157–34.843), and patients in the palliative surgery subgroup had an MST of 12 months (95% CI 7.617–16.383). There was no significant difference between the lobectomy subgroup and the sub-lobectomy subgroup (log-rank p = 0.915), but the survival of the lobectomy and sub-lobectomy subgroups was significantly better than that of the palliative surgery subgroup (log-rank p = 0.001 and p < 0.01, respectively). However, age, gender, and number of chemotherapy cycles had no effect on survival, which was evidenced by the lack of statistically significant differences (**Figure 4**, **Table 6**).

## Discussion

According to the 7th edition of the International Lung Cancer TNM staging standard, the M stage of NSCLC with MPE is defined as M1a (11). The prognosis of such patients is generally considered very poor, and some scholars do not recommend surgical treatment (12). The International Association for the Study of Lung Cancer reports that the MST of cancer patients with MPE is approximately five to eight months, and the five-year survival rate is less than 2% (11, 13). Therefore, NSCLC with MPE belongs to stage IV lung cancer in the latest staging system published by the Union for International Cancer Control (14). Some studies have pointed out that MPE is caused by tumor-induced angiogenesis, and some scholars have also pointed out that vascular endothelial growth factor may be an important cause of MPE by increasing vascular endothelial permeability and exudation (15). Some studies have shown that there are more than 1.5 million new patients with pleural effusion each year in the United States, and MPE caused by cancer accounts for the vast majority of such patients (16, 17). A clinical study involving 1783 patients with MPE found that bronchial lung cancer, particularly lung adenocarcinoma, accounted for disease in 36% of cases (18). There are still no clear guidelines for the treatment of such patients, and there is no confirmed best treatment plan. At present, commonly used clinical treatment methods include continuous therapeutic thoracentesis, chemical pleurodesis, pleural cavity catheter drainage, intrapleural chemotherapy, thoracoscopic pleurodesis and fixation, anti-angiogenesis therapy, molecular targeted therapy, etc. Continuous therapeutic thoracentesis can quickly relieve the clinical symptoms of patients, but the effect is not lasting. It has been reported that 97% of patients with MPE who only undergo continuous thoracentesis will relapse within one month (the average control time is approximately 4.2 days) (19). Spiegler PA et al. reported that the success rate of pleural fixation was approximately 79%, and no recurrence occurred after one month (20). Another study compared the two methods of immediate injection of sclerosing agent after catheter drainage and the injection of sclerosing agent after the drainage volume was less than 150 ml per day. The success rate of both methods of pleural fixation was approximately 79%, which was less than that of the former method only involved the injection of sclerosing agent before hospitalization days (21). With the development of minimally invasive surgical technology, thoracoscopic pleurodesis has been increasingly used by surgeons to control MPE. Several studies have shown that the success rate of thoracoscopic pleurodesis is higher than that after thoracentesis or catheterization, which is approximately 71%–95% (22–24). In our study, thoracoscopic surgery for pleurodesis had an effective rate of 94.8%, which was higher than the 70.7% from thoracic drainage, and according to the CMH X^2^ test, the difference between the two groups was statistically significant. Thus, the effect of VATS on pleural effusion control was better than that of patients who received thoracic drainage.

For NSCLC patients with MPE, the commonly used therapies were thoracentesis, thoracic catheterization, thoracoscopic or thoracotomy, chemotherapy, radiotherapy, molecular targeted therapy, and other adjuvant treatment. A study from Taiwan included 27 NSCLC patients with MPE from 1998 to 2000. These patients received both intrapleural and systemic chemotherapy with standard gemcitabine plus cisplatin regimen followed by radiotherapy, finally followed by three to six cycles of docetaxel monotherapy. The results showed that the RR was 55%, of whom 7% of patients achieved CR. The median progression-free survival and OS were 8 and 16 months, respectively. The one-year survival rate was 63% (95% CI 45%–80%) (25). In another study from Korea, 40 NSCLC patients with MPE received intrapleural and systemic chemotherapy with cytarabine and cisplatin. The results showed that 86.5% of the patients achieved complete remission and 10.8% achieved partial remission. The overall effective rate was 97.3%. The median remission time was 12 months, and that of two patients was nearly 23 months (26). Tieqin Liu reported in his study that 58 patients with M1a NSCLC with MPE or mural pleural metastasis but without distant organ metastasis underwent primary tumor resection (lobectomy or local resection), mediastinal and intrapulmonary lymph node dissection, intrapleural perfusion chemotherapy, and four to six cycles of platinum-containing chemotherapy after surgery. The results showed that the MST of all patients was 34.3 months, and the five-year survival rate was 12.5%. The five-year survival rate of patients with adenocarcinoma was better than that of patients with other pathological types (32.3% vs 25.4%). The five-year survival rate of patients without a smoking history was significantly higher than that of patients with smoking (40.3% vs 18.6%). The five-year survival rate of patients receiving adjuvant chemotherapy after surgery was better than that of patients without chemotherapy (47% vs 23.1%). Additionally, the five-year survival rate of patients receiving local resection was better than that of patients receiving complete resection (31.4% vs 16.3%) (27). A study conducted by Yasuhiko-ohta et al. included 42 patients with NSCLC with a median age of 63.5 years. All patients were diagnosed with pleural metastasis (M1a). Twenty patients underwent pulmonary wedge resection and pleural resection and pleurodesis, two patients received segmentectomy + pleurotomy and pleurodesis, and nineteen patients received lobectomy + pleurotomy and pleurodesis. The survival analysis showed that the distant metastasis were the only factors affecting the survival of all patients (28).

In our study, 96 patients received surgical treatment, which included either partial or complete resection of the primary tumor and metastasis. All patients received systemic chemotherapy. The 195 patients had an MST of 16 months (95% CI 13.439–18.561), which was similar to the results of previous studies. The MST of patients who underwent surgery was 25 months (95% CI 22.373–27.627), which was better than the MST of patients who received thoracic drainage (11 months, 95% CI 9.978–12.022). The MST of patients who received thoracic drainage was similar to that reported in many previous studies. The one-year and three-year survival rates of the VATS group were 88.6% and 21.6%, respectively, which were better than the respective rates of the thoracic drainage group (36.4% and 1%).

The OS of the patients in the VATS group was statistically analyzed in our study. The survival time of patients receiving lobectomy + pleurodesis (p < 0.01) or sub-lobectomy + pleurodesis (p = 0.001) was significantly better than that of patients receiving only pleurodesis. However, there was no significant difference in survival between lobectomy and sub-lobectomy (p = 0.915). Ohta et al. reported that the three-year survival rate of 42 patients with stage M1a lung cancer who received primary tumor and pleural metastasis was 31.4%, and the MST was 17 months (28). Lida et al. also found that 313 lung cancer patients with only mural pleural metastasis had a five-year survival rate of 29.3% and an MST of 34 months. In that study, 256 patients (81.8%) underwent primary tumor resection, and 152 patients (48.6%) underwent resection of all visible pleural metastases. The five-year survival rates of the two groups were 33.1% and 37.1%, respectively (29). NSCLC with MPE had a worse prognosis and shorter survival time than that with pleural metastasis but without MPE. A clinical study involving 98 patients showed that the survival time of lung cancer patients with MPE was significantly shorter than that of lung cancer patients with only pleural metastasis but without MPE. The MST was 38 vs 13 months in those two groups (30). Therefore, the resection of as many tumor tissues as possible seems to provide better survival benefits. The other two studies also provide a theoretical basis for surgical intervention of lung cancer with pleural metastasis, which is a special type of advanced lung cancer (31, 32). This is similar to the results in our study. The surgical resection of the primary tumor and visible metastasis simultaneously provided better survival benefits than resection of the primary tumor or resection of the pleural metastasis only (HR 0.637, 95% CI 0.409–0.993, p < 0.01). However, there was no significant difference between sub-lobectomy and lobectomy. The underlying reason may be that surgery reduces the tumor burden as much as possible without increasing the risk of death, but additional lobectomy does not provide an OS benefit, similar to early-stage NSCLC. However, it increases the risk of surgery and the potential for a poor pleurodesis effect due to the excessive lung tissue loss. Whether other surgical options, such as lymphadenectomy or total pleural resection, can benefit patient survival remains controversial, and larger sample size trials are necessary to provide theoretical evidence for the optimal surgical model for such patients. As a retrospective clinical study, this study also has some limitations, such as selective bias, which results in not all factors being equal between the two study groups, such as the ECOG score. There was a significantly higher proportion of patients with an ECOG score of 0–1 in the VATS group than in the thoracic drainage group, which may be due to the fact that patients in a good general condition are often selected for surgical operations whereas patients with a poor general condition usually choose a drug treatment with less trauma.

## Conclusion

The results of this study showed that VATS was more effective in controlling MPE than thoracic drainage. VATS can significantly improve the OS of patients with advanced lung adenocarcinoma with MPE compared with traditional thoracic drainage methods. Patients who received a lobectomy or sub-lobectomy plus pleurodesis under VATS had a better OS than those who received only pleurodesis under VATS. No smoking history and an ECOG score of 0–1 also improved the OS of these patients.

## Data Availability Statement

The original contributions presented in the study are included in the article/supplementary material. Further inquiries can be directed to the corresponding authors.

## Ethics Statement

The studies involving human participants were reviewed and approved by Review Board of Tianjin Medical University General Hospital. The patients/participants provided their written informed consent to participate in this study.

## Author Contributions

JC, XL, and HL wrote the manuscript. JHL, MD, CX, HZ, SX, SW, ZS, and GC collected the data. ML, JSL, and XL analyzed the data. JC and HL supervised the research. All authors contributed to the article and approved the submitted version.

## Funding

This study was supported by grants from the National Natural Science Foundation of China (82072595, 81773207 and 61973232), Natural Science Foundation of Tianjin (17YFZCSY00840, 18PTZWHZ00240, 19YFZCSY00040, and 19JCYBJC27000), Special Support Program for the High Tech Leader and Team of Tianjin (TJTZJH-GCCCXCYTD-2-6) and Tianjin Key Medical Discipline (Specialty) Construction Project. Funding sources had no role in study design, data collection, and analysis; in the decision to publish; or in the preparation of the manuscript.

## Conflict of Interest

The authors declare that the research was conducted in the absence of any commercial or financial relationships that could be construed as a potential conflict of interest.

## Publisher’s Note

All claims expressed in this article are solely those of the authors and do not necessarily represent those of their affiliated organizations, or those of the publisher, the editors and the reviewers. Any product that may be evaluated in this article, or claim that may be made by its manufacturer, is not guaranteed or endorsed by the publisher.
